# Evaluation of the malaria surveillance system – Adaklu District, Volta Region, Ghana, 2019

**DOI:** 10.1016/j.puhip.2023.100414

**Published:** 2023-07-29

**Authors:** E.E. Agbemafle, C. Kubio, D. Bandoh, M.A. Odikro, C.K. Azagba, R.G. Issahaku, S.O. Sackey

**Affiliations:** a Ghana Field Epidemiology and Laboratory Training Programme, Department of Epidemiology and Disease Control, School of Public Health, College of Health and Allied Sciences, University of Ghana, Legon, Ghana; b Adaklu District Health Directorate, Ghana Health Service, Volta Region, Ghana; c Savannah Regional Health Directorate, Ghana Health Service, Damongo, Ghana

**Keywords:** Malaria, Surveillance system, Evaluation, Adaklu, Volta region, Ghana

## Abstract

**Objectives:**

We evaluated the malaria surveillance system in Adaklu District of the Volta Region of Ghana to determine if the system was meeting its objectives and assessed its usefulness and attributes.

**Study design:**

Descriptive cross-sectional design was used in evaluating the surveillance system.

**Methods:**

We interviewed stakeholders using a semi-structured questionnaire on case detection and reporting. We assessed the system attributes using the Centers for Disease Control and Prevention updated guidelines for evaluating public health surveillance systems. We extracted and reviewed malaria surveillance data from the District Health Management Information System 2. Summary statistics and direct content analysis were performed on quantitative and qualitative data respectively.

**Results:**

Of the 80,441 suspected malaria cases recorded in Adaklu District from 2014 to 2018, 47,917 (59.6%) cases were confirmed. The system was meeting its objective of detecting malaria cases and monitoring trends in the population however, the system missed an epidemic in August 2016. Data generated from the surveillance system is used by the NMCP to aid in the distribution of logistics such as LLINs, RDT test kits, and track malaria control progress in the district. Staff at all levels were able to detect, confirm, treat and report malaria. All sub-districts/health facilities reported to the district and reports were all accurate and timely. The predictive value positive was 62.9%.

**Conclusions:**

The malaria surveillance system in Adaklu District was useful and meeting its set objective of monitoring trends of malaria in the population. It was simple, flexible, acceptable and representative; however, the system was not detecting epidemics. The District Health Management Team should set alert and epidemic thresholds to help detect promptly epidemics of malaria in the district.

## 1 Introduction

In 2018, an estimated 228 million cases of malaria occurred worldwide. Most malaria cases 93% (213 million) occurred in the WHO African Region. Nineteen countries in sub-Saharan Africa and India accounts for almost 85% of the global malaria burden [1]. Ghana recorded approximately 10.2 million suspected malaria cases in the Outpatient Department (OPD) in 2018 [1]. Averagely 27,978 suspected malaria cases were recorded daily. Out of 599 malaria deaths recorded in 2018, 327 (54.6%) were recorded among children under five years [2].

Over the last two decades, the Ghana National Malaria Control Programme (NMCP) has scaled up proven malaria control methods such as the distribution of insecticide-treated nets, sentinel site monitoring, indoor residual spraying, seasonal malaria chemoprevention, intermittent preventive treatment in pregnancy, case management, social and behavioral change communication and vaccination of children 6 months to 2 years amongst others [3]. This attempt is aimed at prevention and control of malaria across the country. Despite the efforts made over the years towards the control of malaria countrywide, the number of cases remain high, hence the need for continuous malaria surveillance system. The malaria surveillance system was set up to systematically monitor trends of malaria in the population, rapidly detect unusual events and epidemics and to plan and set priorities for health policies and programmes. With the setup and running of the surveillance system, it is expected that periodic evaluation of the system is done to ensure its efficient operation and ultimately, improvement in the health status of populations. Ghana adopted the Integrated Disease Surveillance and Response (IDSR) guidelines in 2002, and data on priority diseases are reported through the District Health Information Management System (DHIMS) since 2012. From 2014 to 2016, Adaklu district recorded a total of 26,889 cases of malaria. an average of 8,963 per each of these years. However, 10,987 malaria cases were recorded in 2017 alone, an excess of 2,024 over the previous averages [4]. This trend of events prompted interest regarding the operation of the malaria surveillance system in the district. We evaluated the malaria surveillance system to determine if it is meeting its objectives and also assessed the system's attributes and usefulness.

## 2 Materials and methods

### 2.1 Study design

The malaria surveillance system in Adaklu was evaluated by retrospectively reviewing malaria data from 2014 to 2018. We assessed the usefulness of the surveillance system as well as the system attributes using the Centers for Disease Control and Prevention (CDC) updated guidelines for evaluating public health surveillance systems. The evaluation was conducted within six weeks, January 9th to February 16th, 2019.

### 2.2 Study area

The evaluation was conducted in the Adaklu District in the Volta region of Ghana. Adaklu District is one of the 25 administrative districts in the Volta Region. It shares boundaries with Ho-West to the east, North-Tongu District to the south, Agotime-Ziope District to the north, and Akatsi-North District to the west. There are four sub-districts namely Waya, Ahunda, Helekpe, and Sofa which is the catchment area of the surveillance system in the district. Based on the 2010 Population and Housing Census, Adaklu District has a total projected population of 43,311 with an estimated growth rate of 2.5% per annum [5]. There are 89 communities excluding numerous farming communities and Fulani villages (nomadic populations). The localities in the district are completely rural with no urban settlements. The rainfall pattern in the district is characterized by two rainy seasons commonly referred to as the major and minor seasons. The major season starts from mid-March to July while the minor from August to November [5].

### 2.3 Health care setting

With regards to the health of the people, the government of Ghana through the Ghana Health Service is the main healthcare service provider and supported by the Christian Health Association of Ghana. Although the district lacks a hospital, it has fifteen (15) other health facilities made up of four (5) Health Centers and ten (10) Community Health Planning Services (CHPS) Compounds. The district's epidemiological profile shows a concurrent significant prevalence of diseases including malaria, upper respiratory tract infections, intestinal worms, diarrhoea, and rheumatism/joint pains [5].

Before the introduction of the CHPS concept in mainly rural areas in Ghana, the Health Center was the first point of contact between the formal health delivery system and the client. The facility is headed by a Medical/Physician assistant. These facilities provide basic curative and preventive services to both adults and children, mainly medical, midwifery, laboratory, public health and nutritional services on out-patient bases i.e., these facilities only detain patients for observation and patients who require admission are referred to hospitals [6]. The CHPS compound/zones are designed to deliver community level health promotion, prevention and primary clinical care services to communities while supported by a system. Services provided at the CHPS compound include antenatal care (ANC), postnatal care (PNC) through community health officers (CHOs) and emergency delivery as well as treatment of minor ailments. These two types of health facilities i.e., Health Center and CHPS compound by nature of their structure and operations with regards to infrastructure, equipment and personnel do not have the capacity to treat severe and complicated forms of illnesses, these cases are therefore referred to hospitals in Ho and Adidome for treatment/management [7].

### 2.4 Population characteristics

The population under malaria surveillance includes the general population, however, the most at-risk group of people are children under five years and pregnant women. The stakeholders involved in the operations of the system include the community health management committee members (CHMC), District Director of Health Services, malaria focal persons, health information officers, surveillance officers, prescribers, nurses, midwives, laboratorians and community-based surveillance volunteers in Adaklu District.

### 2.5 Data collection

We extracted secondary data on malaria morbidity and mortality for Adaklu District, from the DHIMS 2 platform from January 2014 to December 2018.

We conducted key informant interviews, involving the regional and district malaria focal persons on the malaria surveillance system. A total of twelve health care workers, four at the Regional and District Health Directorate, and eight (8) staff at the facility level were interviewed. We interviewed these personnel on their knowledge and involvement in the surveillance system and also partook in surveillance activities while observing practices. These interviews focused on malaria case definitions, flow chart for malaria surveillance, data collection, analysis, dissemination, and feedback at the district, regional and national levels. We reviewed records on monthly outpatient morbidity return forms, NMCP antimalarial reports, and Integrated Disease Surveillance and Response (IDSR) returns on malaria. System usefulness and attributes were evaluated using CDC updated guidelines for evaluating public health surveillance [8] Methods used in assessing the objectives, usefulness and system attributes are illustrated on Table 1.

**Table 1:** Methods and parameters used in evaluating the objectives, usefulness and attributes of the malaria surveillance system, Adaklu District, Volta Region, Ghana-2019.

Objective		Method	Parameters evaluated
1. To systematically monitor trends of malaria in the population		Observation and records review	Availability of information on trend analysis (graphs in reports, display on notice boards)
2. To rapidly detect unusual events or epidemics		Observation records review and data analysis	Availability of information on detected epidemics (threshold calculations)
3. To plan and set priorities for health policies and programmes		Observation and records review	Documentation on policies put in place based on surveillance data
**Usefulness**			
Usefulness of the surveillance system	**Method**	**Indicators evaluated** - Identification of vulnerable groups - Establishment of thresholds - Monitoring morbidity trends - Use of data for action (control and prevention)	
	Records review interview		
**System attributes**			
**Attribute**	**Method**	**Indicators evaluated**	
Simplicity	Interview Observations Records review	- Staff knowledge on case definition - Simple case definition - Ease of confirming a case through investigation - Clarity and ease of completion of surveillance forms - Knowledge on reporting channels - Time spent and collecting a reporting data	
Acceptability	Interview, records review	- Proportion of sub-districts reporting per surveillance period - Mean time from case detection to lab testing - Proportion of surveillance officers interviewed willing to participate in surveillance system	
Stability	Interview Assessment of logistics and equipment	- Frequency of system operation interruptions - Number of unscheduled computer system outages - Availability of backup and storage facilities - Availability of RDTs - Breakdown of microscopes - Donor support	
Flexibility	Interview, Records review	- Use of case definition in detecting other conditions - Changes in operations - integration of malaria with other morbidities	
Data Quality	Records review	- Percentage of completed monthly data returns form - Completeness of consulting room registers	
Timeliness	Data review	- Proportion of health facilities submitting monthly reports to the DHD by 5th of the ensuing month	
Representativeness	Data review	- Proportion of health facilities reporting per month - Variability in age and sex distribution of cases	
Predictive value positive (pvp)	Data review and analysis	- Proportion of suspected cases testing positive 2014 to 2018 - Pvp = confirmed cases/suspected cases *100	

### 2.6 Data analysis

Qualitative and quantitative data analysis approach was adopted in analyzing data. We performed summary descriptive statistics on quantitative malaria data extracted from DHIMS 2 covering 2014 to 2018 and directed content analysis on qualitative data obtained through interviews. The analysis was done using Microsoft Excel 2013. Five-year morbidity trends against thresholds were presented in a graph, and other results were presented in text, tables and graphs.

## 3 Results

### 3.1 Overview

Of the twelve stakeholders interviewed, four were females. The mean age of respondents was 29.5 ± 5.1 years. All staff interviewed admitted that they were involved in the malaria surveillance system and understood that data collected will be useful in decision making if analyzed and disseminated to stakeholders.

### 3.2 Malaria morbidity surveillance data

Adaklu District recorded a total of 80,441 suspected malaria cases from 2014 to 2018. Of the total number of cases suspected, 47,917 (59.6%) were confirmed positive, 19,226 (40%) cases were recorded among children under five years, 859 (1.8%) among pregnant women and one severe malaria case was confirmed in 2016.

### 3.3 Case definition

Suspected uncomplicated malaria is any person with fever or history of fever within 24 h; without signs of severe disease (vital organ dysfunction). Suspected severe malaria is any patient hospitalized with severe febrile disease with accompanying vital organ dysfunction diagnosed clinically. A confirmed case of malaria is the occurrence of malaria illness/disease in a person in whom the presence of malaria parasites in the blood has been confirmed by parasitological testing [9].

The malaria case definition was found in the IDSR guidelines at the regional and district levels. However, there was no case definition of malaria at other levels of the surveillance system; facilities only relied on case management protocols that had signs and symptoms written on them as a guide to diagnosing and managing malaria cases.

### 3.4 Operations of the malaria surveillance system in Adaklu District

The malaria surveillance system in Adaklu District is passive, integrated with other disease conditions and events under surveillance, and every individual in the district is under surveillance for malaria. In the district, diagnosis of malaria is mainly done through RDT. Confirmed cases of uncomplicated malaria are treated in the health facilities while severe malaria cases are referred to Ho or Adidome Hospitals. The main referral hospitals are Volta Regional Hospital, Ho, Municipal Hospital Ho, and Adidome Government Hospital.

Data on uncomplicated malaria is collected and reported on three different forms: the monthly outpatient morbidity returns, monthly malaria data returns on antimalarials, and NMCP antenatal/maternity monthly data returns. Data in the consulting room registers are entered into these forms and reported into DHIMS 2 on monthly bases. The monthly malaria data returns collect data on antimalarials, the number of out-patient treated with malaria whether tested or not, and data on drugs and other commodities. In this register, data is collected per age as children under five years and people five years and above. For the NMCP antenatal/maternity monthly data returns, data is collected on antenatal and maternity records. Attendance of pregnant women and number of IPTP (sulphadoxine pyrimethamine) administered to pregnant women as well as stock of the medication in the health facility.

Health facilities compile two copies of the monthly reports, a copy is kept in the facility, and the other is submitted to the DHMT. Paper-based copies of completed reports are kept in cupboards in the health facilities under lock and key and only authorized persons including physician assistants, nurses have access to such forms. Each health facility reports to the District Health Directorate (DHD). At the district level, data is entered into DHIMS, once the data is entered all other levels of the surveillance system can access the data electronically. Upon receipt of the paper-based reporting forms, the date and time of receipt of data are written on the reporting forms to track late submission. To ensure data quality, data validation is done at each level of the surveillance system. From the sub-district to the regional levels of the surveillance system, a team of health workers validates the data by comparing data from the source documents (consulting room register) to the reporting forms (monthly outpatient morbidity returns, monthly malaria data returns on antimalarials and NMCP antenatal/maternity monthly data returns). From the regional level to the lower levels of the system, feedback is sent about the accuracy and validity of data. Where issues of data quality arose, it was traced to the level where the errors were made and such is amended.

Data analysis was done at the district and regional levels by Disease Control and Health Information Officers using Microsoft Excel. The result of morbidity trends was displayed in their offices and presentations were done during mid-year and annual performance reviews at the district, regional and national levels. Apart from the Ghana Health Service (GHS), NMCP, quarterly, half-yearly and annual report were shared with Christian Health Association of Ghana (CHAG) and Non-Governmental Organizations such as Good Neighbors, Plan International Ghana, and Marie Stopes. These are NGOs who are interested in malaria surveillance in the district. Dissemination of information was done across all levels.

### 3.5 Data communication in the malaria surveillance system

**Fig. 1:**
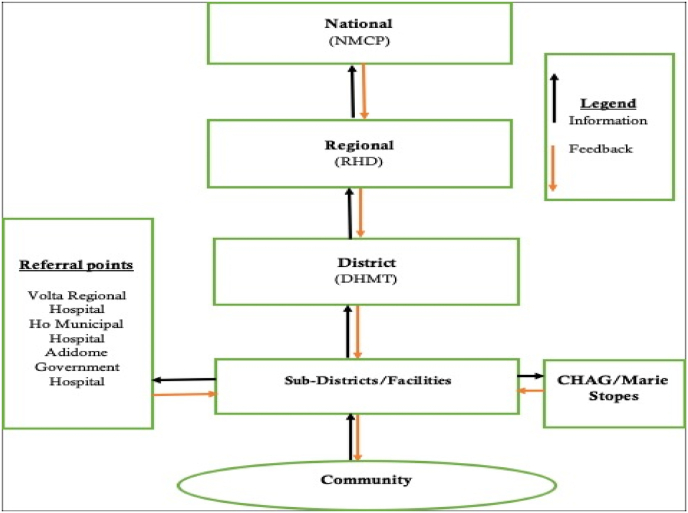
Data communication flowchart in the malaria surveillance system, Adaklu District, Volta Region, Ghana-2019.

There was no data flow chart of the surveillance system at the various levels. However, health care workers verbalized that there were five (5) levels of reporting sources within the malaria surveillance system. Data flows upwards from the community level to the national level and feedback is given from the national level as well as other partners (local or international) downwards as depicted in Fig. 1.

### 3.6 Objectives of the surveillance system

#### 3.6.1 **Objective 1:** To systematically monitor trends of malaria in the population

Trends of malaria morbidity were systematically monitored in the population. The highest number of malaria cases for most of the years within the period of evaluation occurred in August for 2014, 2016, and 2017. However, 2015 and 2018 recorded their highest cases in July. The year 2018 recorded the highest number of cases compared to the other years. The district displayed graphs depicting trends of malaria on their notice boards and in their annual reports and also made presentation during their mid-year and annual reviews.

#### 3.6.2 **Objective 2:** To rapidly detect unusual events or epidemics

**Fig. 2:**
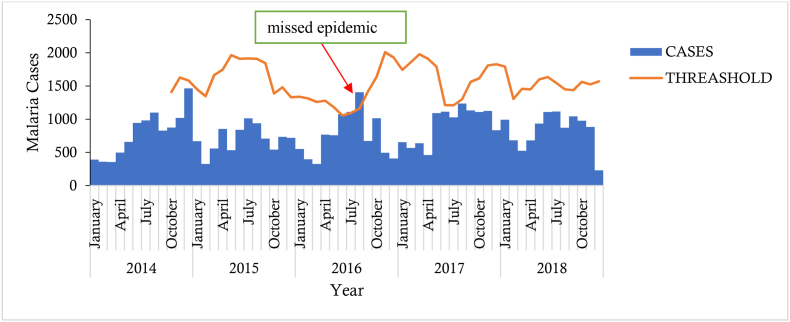
Malaria cases against a threshold in Adaklu District, Volta Region, Ghana, 2014-2018.

The district was not able to detect any epidemic over the period under review, 2014 to 2018. However, threshold calculations done by the evaluation team using the Cumulative Sum (CuSum) method based on previous data indicated that there was a missed epidemic in August 2018. The epidemic was not detected by the system because there was no threshold calculation for the disease (alert and epidemic thresholds) as shown in Fig. 2.

#### 3.6.3 **Objective 3:** To plan and set priorities for health policies and programmes

At the district level, there was the engagement of the community health management committee (CHMC) members in the malaria control and prevention campaigns in the communities. At the regional level, information from the system was used to aid the prioritization and selection of sentinel sites for malaria in pregnancy surveillance.

### 3.7 The usefulness of the malaria surveillance system

The malaria surveillance system was used to monitor morbidity trends at all levels and also evaluate control and preventive measures. The data from the system is used by the NMCP to aid in the distribution of logistics such as LLINs, RDT test kits, and track malaria control progress in the district. Refresher training on case management was routinely conducted for health workers in the sub-District/facilities. The following quotes from the malaria focal person reiterates this finding;

“Data from the malaria surveillance system has been very useful in knowing the areas that have more malaria cases and so specific interventions are carried out such as LLINs distribution for preventive purposes and RDT test kits for diagnostic purposes. Also, the NMCP has been involved in organizing case management workshops for staff to ensure proper management of malaria cases”.

### 3.8 System attributes

*Simplicity:* All eight (8) health care workers interviewed at the facility level could mention at least three (3) signs and symptoms of malaria representing 100%. Although there was no poster of malaria case definition on notice boards or walls of all the facilities visited, other malaria protocols were displayed on the walls and flip charts for malaria case management. Hence there was no difficulty in diagnosing malaria cases. The surveillance forms (the monthly outpatient morbidity returns, monthly malaria data returns on antimalarials, and NMCP antenatal/maternity monthly data returns) were clear and easy to complete with regards to what data was needed to be collected, making data collection easy. Confirmation of suspected malaria cases was done with malaria RDT kit. This took between 15 and 20 min to get results. On a monthly basis, time spent on compiling, collating, maintaining, and reporting data was said to be averagely 3 h. The following quote from a health facility surveillance focal person reiterates this finding;

“I think malaria surveillance system is simple, I am saying this because once patients talk about their symptom it is easy to suspect malaria, testing with RDT also takes few minutes between 15 and 20 minutes. With regards to reporting, the reporting forms have clear and simple variables to be collected from the registers and it takes about 3 hours to put together the monthly data/report to be reported to the district level”.

*Flexibility:* The malaria morbidity reporting is integrated with other conditions. The major change in the malaria surveillance system was the protocol on testing all suspected cases and only positive cases treated. However, this partially affected the flexibility of the system”. The following quotes from a physician assistant in-charge of Waya Health Center suggests the level of flexibility of the surveillance system;

“The integration of malaria morbidity reporting with other morbidities did not compromise reporting since the system made it possible to report all other cases together. Also, the change in policy i.e., testing of all suspected malaria cases, does not require additional human resources but requires additional testing capacity at the facility level. Meaning that the RDT kits should always be available for testing suspected malaria cases, however, with the occasional shortage in RDT kits, it was difficult to test all suspected malaria cases.

*Data quality*: At the health facility level, data quality was estimated to be 100% complete from all reports sampled from the eight (8) facilities visited. There were no “blank” spaces observed on any of the reporting forms. “Zero” reporting was one key feature observed in the reporting forms. Reporting forms at the DHD, when compared to those at the health facilities, had no disparities although there was difficulty with legibility with regards to the carbon copies. After checking through three (3) years' reports, thus 2016, 2017, and 2018, the 2018 report recorded the highest score for data quality at 100%.

*Timeliness:* The system had 100% on timely reports submission both at the district and regional levels from 2016 to 2018. Monthly reports were expected from fifteen health facilities in the district and all fifteen facilities reported on or before the 5th of every month from January to December 2018. The district was also expected to complete data entry into DHIMS 2 by the 15th of every month and this electronic data can be assessed at the regional and national levels. This was also done at the regional level.

*Acceptability:* Prescribers, nurses, and laboratorians were willing to participate in the malaria surveillance system. This was evidenced by documentation of suspected and confirmed cases in the consulting room and laboratory registers. The 100% timely reporting rate also highlighted acceptability and monthly feedback from the district was given to the sub-districts and facilities.

*Stability:* The system was able to collect, collate, manage, and provide data properly without failure although there was an occasional shortage of RDT kits and power outages. Until 2017, there were occasional shortages of RDT kits. There was no backup in place in terms of power outages and staff had to wait till the main power source was restored before they could enter and manage data in the DHIMS 2, but these did not compromise the stability of the system. The NMCP and other stakeholders also conducted routine training of healthcare workers on malaria case management aiming at improving malaria case management, surveillance and also supported logistic supply of RDTs, Reagents, LLINs etc. to the district. The following quotes summarizes these findings;

The main challenges we have is shortages of RDT kits sometimes and power outages especially during the “dumsor” era (the period of rampant electricity outages in Ghana, 2014-2016). In times when we didn’t have RDT kits, we only treated patients presenting with fever, headache and other symptoms as malaria without any form of confirmation. Regarding power outages, anytime we were working on the computer and the light went off, we had no option than to wait till the lights come back before we can continue working on our data because there are no electricity generators to provide alternative source of electricity for managing electronic data.

**Fig. 3:**
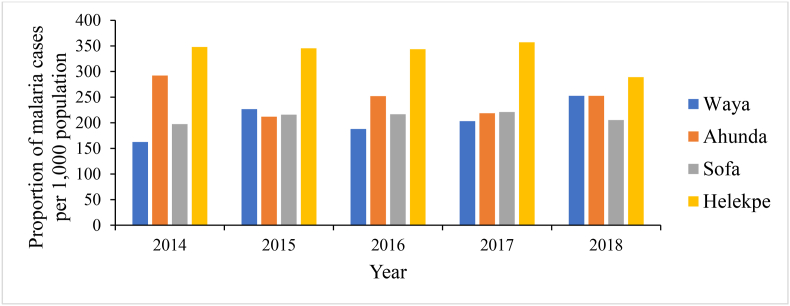
Malaria cases reported by Sub-Districts in Adaklu District, Volta Region, Ghana, 2014-2018.

*Representativeness:* All fifteen health facilities in the four sub-districts submitted monthly malaria reports to the district, Helepke sub-district recorded the highest proportion of cases per 1,000 population during the five years. Reporting forms captured malaria in children under five (5) years, above five, and pregnant women. Cases were detected all year round. This is highlighted in Fig. 3.

*Predictive value positive:* The estimated pvp for the five (5) year period was 62.7% (47,917/76,386). The proportion of people identified as cases in the surveillance system who truly had malaria was 62.7%.

## 4 Discussion

The malaria surveillance system in the Adaklu District is achieving its surveillance objectives of systematically monitoring trends of the disease in the population; however, epidemics could not be detected because there were no threshold calculations over the years. This is evidenced by the system's inability to detect an epidemic which went unnoticed in August 2016. Threshold setting and monitoring is very important in ensuring that epidemics are prevented and otherwise quickly noticed and controlled.

The peak periods of confirmed malaria cases in the district were between March and October, where about 60% of cases were recorded during the five years. This depicted a seasonal trend of cases taking into consideration the proportion of cases recorded between March and October, which happens to be a period corresponding to the rainy seasons in Ghana. Similar observations were made by other studies that reported significantly higher malaria cases during the rainy seasons [[10], [11], [12], [13]]. This trend can partly be attributed to the fact that mosquito breeding sites are rampant during the rainy season, and with time, these mosquitoes mature into adulthood. As they bite humans, the infection is transmitted, signs and symptoms development and confirmation of the parasites is evidence of infection. Also, this recurrent trend suggests that interventions such as indoor residual spraying (IRS), use of ITNs, and other preventive measures may not have been appropriately targeted in curbing the situation during those peak periods.

The surveillance forms (the monthly outpatient morbidity returns, monthly malaria data returns on antimalarials, and NMCP antenatal/maternity monthly data returns) were easy to complete, and the diagnosis was mostly by RDT. This indicated a simple malaria surveillance system in the Adaklu District. This finding is consistent with the evaluation conducted in Ebonyi state Nigeria [13], where 80% of stakeholders interviewed reported that the forms were easy to complete.

The system was found to be flexible, acceptable by stakeholders, and timely, 100% timeliness of reporting by all health facilities in the district emphasized acceptability unlike a similar malaria surveillance system evaluation in Kaduna Nigeria which recorded timeliness to be 37.7% [14]. The high acceptability and timeliness of the system could be attributed to stakeholder interest in the surveillance system.

The system was relatively stable as it could collect, collate, manage, and provide data properly without failure despite occasional power outages and a shortage of RDT kits. This finding is consistent with findings from the evaluation of Dengue fever surveillance system in Taiwan [15] and Meningitis system evaluation in the Yendi Municipality in the Northern Region of Ghana [16]. They attributed these achievements to early detection and response to any unforeseen occurrences.

Data quality was high at 95%, the data was devoid of incomplete or blank spaces. One significant feature on the reporting forms in Adaklu District was “Zero reporting”. This can be attributed to the high level of acceptability of stakeholders involved in the system. The flexible nature of the system made it possible for the system to adapt to a change from treating suspected malaria cases without confirmation to treating only confirmed positive cases without significant stress on the system with regards to resources. This is because RDT did not require high technical expertise to perform and produced results. The utilization of the RDT kit for malaria diagnosis in the District is consistent with the finding in a similar evaluation conducted in Ebonyi state Nigeria [13]. The authors of this evaluation indicated that RDT or microscopy was mainly used for testing for the presence of malaria parasites in Ebonyi state. The system is representative in terms of person, place, and time hence the reliability of the data being generated by the system. Malaria cases were reported from all sub-districts and covered males, females, children under five years, persons five years or older and pregnant women.

The functional referral system in the district ensured that suspected or confirmed severe malaria cases were referred to any of the following hospitals, Ho Municipal Hospital, Volta Regional Hospital, or Adidome Hospital which can admit and timely treat severe malaria cases.

## 5 Limitations

The review required that interviewees recalled events over the past five years, there is, therefore, the likelihood of recall bias. However, this was minimized since frontline workers who are directly involved in the surveillance system were interviewed.

## 6 Conclusions and recommendations

The malaria surveillance system operated by the Adaklu District Health Directorate was useful and achieving most of its objectives except the ability to detect epidemics. The system was found to be simple, flexible, fairly stable, acceptable, and representative. Timeliness and data quality were good and PVP was high. Monthly feedbacks, although verbal, from the district, was given to the sub-Districts and facilities.

We, therefore, recommend that the DHMT should set alert and epidemic thresholds for malaria cases to help detect promptly epidemics of malaria in the district. The Regional Health Information and Surveillance Officers should ensure that feedbacks at all levels of the surveillance system are documented. The Regional and District Disease Surveillance officers should provide malaria case definitions and surveillance communication flow charts across all levels of the surveillance system and ensure routine in-service training of staff on the IDSR.

## Public health action

We assisted the DHMT to calculate thresholds for malaria epidemic detection and drew a data communication flow chart for the surveillance system in the district.

## Funding

The evaluation did not receive any specific grant from funding agencies in the public, private, commercial or non-profit-organization.

## Ethical consideration

Ghana Field Epidemiology and Laboratory Training Program (GFELTP of the University of Ghana) sought permission from the Ghana Health Service for this evaluation. This was done through the Regional and District Directors of Health Services as well as the National Malaria Control Programme (NMCP). A written letter was approved, and introductory letters from the respective offices were given for the evaluation to be carried out. Key informants we interviewed gave informed written consent before granting interviews**.**

## Declaration of competing interest

Authors declare that there is no competing interest as far as this work is concerned. All views expressed in the piece of work are views of the authors and does not represent the views of position of any organization.

## References

[1] WHO (2018).

[2] USAID and CDC, “U . S . President ’ s Malaria Initiative Cambodia: Malaria Operational Plan FY 2020,” Cambodia, 2020. [Online]. Available: https://www.pmi.gov/docs/default-source/default-document-library/malaria-operational-plans/fy20/fy-2020-ghana-malaria-operational-plan.pdf?sfvrsn=6.

[3] U.S (2019). https://www.pmi.gov/docs/default-source/default-document-library/malaria-operational-plans/fy19/fy-2019-ghana-abbreviated-malaria-operational-plan.pdf?sfvrsn=5.

[4] DHMT (2019).

[5] Ghana Statistical Service (2014). https://new-ndpc-static1.s3.amazonaws.com/CACHES/PUBLICATIONS/2016/06/06/ADAKLU.pdf.

[6] Ghana Statistical Serivce and Ministry of Health Ghana (2002). Overview of the health system in Ghana. Ghana Serv. Provis. Assess. Surv..

[7] Ministry of Health (2014). https://www.moh.gov.gh/wp-content/uploads/2016/02/CHPS-policy-final-working-draft-for-validation.pdf.

[8] US-CDC (2013). Eval-Surv-Sys_Fieldg_Final_09262013. https://www.cdc.gov/globalhealth/healthprotection/fetp/training_modules/12/eval-surv-sys_fieldg_final_09262013.pdf.

[9] U. Ministry of Health (2011). Technical guidelines for integrated disease surveillance and response.

[10] Kipruto E.K. (2017). Effect of climatic variability on malaria trends in Baringo County, Kenya. Malar. J..

[11] Awine T., Malm K., Peprah N.Y., Silal S.P. (2018).

[12] Akhtar Qureshi N., Fatima H., Afzal M., Khattak A.A., Nawaz M.A. (2019). Occurrence and seasonal variation of human Plasmodium infection in Punjab Province, Pakistan. BMC Infect. Dis..

[13] Joseph A., Patrick N., Lawrence N., Lilian O., Olufemi A. (2017).

[14] Ibrahim B.S., Abubakar A.A., Bajoga U.A., Nguku P.M. (2017). Evaluation of the malaria surveillance system in Kaduna state, Nigeria 2016. Online J. Public Health Inform..

[15] Mckerr C., Lo Y., Edeghere O., Bracebridge S. (2015).

[16] Kaburi B.B. (2017).

