# Supplementary material for: Evaluation of the malaria surveillance system – Adaklu District, Volta Region, Ghana, 2019
Source: Public Health Pract (Oxf). 2023 Jul 29;6:100414. doi: 10.1016/j.puhip.2023.100414 (PMC10410592; doi:10.1016/j.puhip.2023.100414)
Supplement: Multimedia component 1 [file mmc1.docx]

### **KEY INFORMANT INTERVIEW GUIDE**

**Malaria surveillance system evaluation in the Ho Regional Health Directorate, Volta region**

Respondent No.: ______________________________

Position: ____________________________________

District: _____________________________________

**A. Purpose and objective of the system**

1. What is the purpose of the Malaria Surveillance System?

2. Do you have a written documentation of the objectives of the Malaria surveillance system?

a. Yes

b. No

c. Don’t know

3. What is the data collected used for?

……………………………………………………………………………………………………

……………………………………………………………………………………………………

……………………………………………………………………………………………………

……………………………………………………………………………………………………

4. Who uses your data?

…………………………………………………………………………………………………..

…………………………………………………………………………………………………..

5. Do you have a list of case definitions available to you?

a. Yes

b. No (skip 4a)

4a. If Yes, from where?

6. Where does your department reside within the Malaria Surveillance system?

……………………………………………………………………………………………………..

……………………………………………………………………………………………………..

7. Do you have a flow chart of the Malaria surveillance system at your level?

a. Yes (if yes, please submit a copy)

b. No

8. Is the Malaria surveillance system at your level integrated with other systems?

a. Yes

b. No

If yes, to what extent?

**B. Components of the system**

1. Can you kindly describe the population under your surveillance as regards to Malaria?

……………………………………………………………………………………………………

……………………………………………………………………………………………………

……………………………………………………………………………………………………

……………………………………………………………………………………………………

……………………………………………………………………………………………………

Hint: projected population for 2018

Number of sub-districts

Number of health facilities and name them

Types of facilities and their location

Community based service providers

2. How frequently do you collect or receive data on Malaria?

…………………………………………………………………………………………………….

3. Who enters that data?

……………………………………………………………………………………………………

……………………………………………………………………………………………………

4. What system of coding is used to enter data?

……………………………………………………………………………………………………

……………………………………………………………………………………………………

5. Is data quality check or validation conducted?

a. Yes

b. No

If yes, how is it done?

…………………………………………………………………………………………………..

……………………………………………………………………………………………………

6. Who does it?

…………………………………………………………………………………………………….

7. Where are your data stored?

……………………………………………………………………………………………………

Is it under lock and key? a. Yes b. No

Do the storage facilities have passwords? a. Yes b. No

8. Do you create “backups” for your data? a. Yes b. No

If yes, how and where?

……………………………………………………………………………………………………..

……………………………………………………………………………………………………..

9. How are your data analysis done? Who does it?

……………………………………………………………………………………………………..

……………………………………………………………………………………………………..

…………………………………………………………………………………………………….

…………………………………………………………………………………………………….

10. To whom do you disseminate your data and how?

…………………………………………………………………………………………………….

…………………………………………………………………………………………………….

……………………………………………………………………………………………………...

11. How do you ensure patient privacy, data confidentiality and system security?

………………………………………………………………………………………………………

………………………………………………………………………………………………………

………………………………………………………………………………………………………

……………………………………………………………………………………………………...

12. Do you use a records management program? A. Yes b. No

If yes, which?

…………………………………………………………………………………………………..

………………………………………………………………………………………………….

**System Attributes**

**A. Simplicity** (structure and ease of operation)

i) What amount and type of data is needed to meet the case definition? (Demographics, behavioral, exposure information)

………………………………………………………………………………………………………………………………………………………………………………………………………………………………………………………………………………………………………………………………………………………………………………………………………………………………

ii) Are there malaria case definitions displayed at the health facilities? Yes No

iii) Are malaria reporting procedures displayed? Yes No

iv) How many reporting sources are there for this system?

……………………………………………………………………………………………………..

v) How many organizations are involved in receiving case reports?

……………………………………………………………………………………………………..

vi) How much time is spent on collecting, maintaining, analyzing and transmitting information?

……………………………………………………………………………………………………..

**B. Flexibility** (ability to adopt to change**)**

i) Has there been any changes in the system (evidence of any past demands)

………………………………………………………………………………………………………………………………………………………………………………………………………………

ii) Why were these changes done?

………………………………………………………………………………………………………………………………………………………………………………………………………………………………………………………………………………………………………………………

**C. Data quality** (completeness and validity of data)

i) What percentage of unknown or blanks responses to items on the surveillance form are there?

……………………………………………………………………………………………………

ii) What measures are in place to ensure completeness and accuracy of data?

………………………………………………………………………………………………………………………………………………………………………………………………………………………………………………………………………………………………………………………………………………………………………………………………………………………………

iii) Suggest recommendations to improve data quality

……………………………………………………………………………………………………………………………………………………………………………………………………………………………………………………………………………………………………………………………………………………………………………………………………………………………....

**D. Acceptability** (willingness of persons or organizations to participate in the system)

i) How many health facilities are in the district?

ii) How many of them report in each year?

iii) Are people who are part of the system e.g. lab scientist and doctors willing to participate in the system. A. Yes b. No

iv) Are patients willing to come to health facility on time? A. Yes b. No

v) What difficulties are there in filling the surveillance forms?

………………………………………………………………………………………………………………………………………………………………………………………………………………

vi) How can willingness to participate be improved?

………………………………………………………………………………………………………………………………………………………………………………………………………………………………………………………………………………………………………………………………………………………………………………………………………………………………

**E. Sensitivity** (Proportion of cases detected by the surveillance system)

i) Visit a health facility and review medical records of cases to find out how many cases were documented for the past year.

ii) Visit DHMT to review documents to identify how many suspected cases were reported

(In relation to visited health facilities visited)

iii) What recent changes if any e.g. diagnostic or surveillance procedures have influenced sensitivity or reporting

iv) Recommendations from facilities on how system could be improved.

**F. Predictive value Positive** (proportion of cases that actually have the disease under surveillance)

i) Are samples being sent for confirmation? A. Yes b. No

ii) Where and how many were confirmed positive

………………………………………………………………………………………………………………………………………………………………………………………………………………

**G.** **Representativeness** (how accurately the system describes the occurrence of the disease in person, place and time)

i) What are the characteristics of the population under surveillance (in relation to age, sex, geographic location, socioeconomic status and access to health care?)

……………………………………………………………………………………………………………………………………………………………………………………………………………………………………………………………………………………………………………………………………………………………………………………………………………………………………………………………………………………………………………………………………...

H. **Stability** (ability to collect, manage and provide data properly without failure)

i) How many unscheduled power outages and breakdown of system computers occur in a year?

………………………………………………………………………………………………………………………………………………………………………………………………………………

ii) What kind of backup is in place in terms of power outages and system computer breakdown?

…………………………………………………………………………………………………….

iii) List the areas that need more support

………………………………………………………………………………………………………………………………………………………………………………………………………………………………………………………………………………………………………………………………………………………………………………………………………………………………

iv) Suggest ways to overcome these challenges

………………………………………………………………………………………………………………………………………………………………………………………………………………………………………………………………………………………………………………………………………………………………………………………………………………………………………………………………………………………………………………………………………

**KNOWLEDGE AND PERFORMANCE OF MALARIA** **SURVEILLANCE SYSTEM IN ADAKLU DISTRICT, VOLTA REGION GHANA**

**HEALTH FACILITY BASE QUESTIONNAIRE**

Q1. Are you aware of the malaria surveillance system? YES NO

Q2. Can you please tell me what the case definition of malaria is?

………………………………………………………………………………………………………………………………………………………………………………………………………………Q3. What are the components of the malaria surveillance case definition ………………………………………………………………………………………………………………………………………………………………………………………………………………Q4. What are the signs and symptoms of malaria ………………………………………………………………………………………………………………………………………………………………………………………………………………

Q5. Do you have a poster on malaria case definition on the walls in your consulting room? YES NO

IF YES poster seen YES/NO

Q6. Do you get feedback from the district health management team on your malaria report? YES NO

IF NO SKIP Q5

Q7. Is the feedback regular and how often?

a. Monthly b. every 3 months c. every 4 months d. every 6 months

e. yearly

Q8. Have you had any training on malaria in the last 2 years? YES NO

IF YES WHAT TRAINING………………………………………………………………………………...

Q9. What are some of the constraints you have with regards to malaria management and reporting

………………………………………………………………………………………………………………………………………………………………………………………………………………………………………………………………………………………………………………

**Reference**

•Using surveillance data and outbreak investigations to strengthen measles immunization

programs, Geneva, World Health Organization. WHO/EPI/GEN/96.02

•WHO Guidelines for Epidemic Preparedness and Response to Measles Outbreaks

WHO/CDS/CSR/ISR/99.1
